# Mental health literacy among primary care providers in Hungary: a vignette-based survey

**DOI:** 10.1186/s12991-024-00539-5

**Published:** 2025-01-28

**Authors:** Valerie S. Swisher, Dorottya Őri, Zoltán Rihmer, Róbert Wernigg

**Affiliations:** 1 The Pennsylvania State University, State College, PA USA; 2 Institute of Behavioural Sciences, Semmelweis University, Budapest, Hungary; 3 Heim Pal National Pediatric Institute, Budapest, Hungary; 4 Nyírő Gyula National Institute for Psychiatry and Addictions, Budapest, Hungary; 5 National Directorate-General for Hospitals, Budapest, Hungary

**Keywords:** Primary care, Anxiety disorders, Stigma, Mental health literacy, Depression, Obsessive-compulsive disorder

## Abstract

**Objective:**

This study examined mental health literacy and predictors of disorder recognition among primary care providers (PCPs) in Hungary.

**Methods:**

208 PCPs in Hungary completed a survey assessing demographics, mental health stigma, and exposure to mental health (i.e., personal experiences and having a family member/friend with a mental health condition). Participants read six vignettes describing obsessive-compulsive disorder (OCD) harm/aggression subtype (OCD-Aggression), OCD order/symmetry subtype (OCD-Order), generalized anxiety disorder (GAD), social anxiety disorder (SAD), panic disorder (PD), and major depressive disorder (MDD) and were asked to identify each condition, perceived disorder causes, and provide treatment referrals. Descriptive analyses were used to characterize disorder recognition rates, perceived disorder causes, and treatment referrals. Binary logistic regression analyses were conducted to examine the degree to which demographic characteristics, mental health stigma, and exposure to mental health conditions predict accurate disorder recognition.

**Results:**

Identification rates for each vignette were: OCD-Aggression (27.9%), OCD-Order (75.5%), SAD (34.1%), GAD (76.0%), PD (78.8%), and MDD (91.3%). First-choice treatment referrals were a psychiatrist for OCD-Aggression (63.0%), OCD-Order (53.8%), and MDD (46.6%), a psychologist/therapist for SAD (58.7%) and GAD (48.6%), and a PCP for PD (39.9%). Mislabeling conditions was significantly associated with older age (for GAD, OCD-Aggression, PD and MDD), male gender (for GAD), greater mental health stigma (for OCD-Order), and lack of exposure to mental health conditions (for SAD).

**Conclusions:**

Findings highlight strengths (e.g., depression recognition) and limitations in knowledge of mental health conditions among PCPs in Hungary and identifies targets to address to improve mental health literacy.

**Supplementary Information:**

The online version contains supplementary material available at 10.1186/s12991-024-00539-5.

## Introduction

Mental illness is a leading cause of global disease burden [1], enacting substantial psychosocial, occupational, and functional impairment. As the majority of people with mental health problems seek and receive mental health care from primary care providers (PCPs) [2, 3], PCPs assume an important role in the identification of mental illness. Despite being the default mental health system in many countries worldwide (e.g [4–6]), the management of mental health conditions in primary care is suboptimal. Recognition rates of common mental health conditions among PCPs vary considerably depending on the condition, ranging from 15 to 96% (e.g [7–11]). As primary care providers (PCPs) are typically the first point of contact for patients and are responsible for coordinating individual care plans, identifying gaps that can be addressed through targeted training is essential for improving patient care.

The mental health knowledge gap among PCPs may be even more pronounced in countries like Hungary, where a post-socialist transition [12] and economic adversities may have hindered growth in mental health care and training [12, 13]. Hungarians are more likely to have negative attitudes and less openness toward depression relative to other countries in Europe [14], and people with mental illness are among the most rejected groups in Hungarian society [15]. Only one known study has examined the identification of mental health conditions among PCPs in Hungary. Comparing PCP diagnoses to diagnoses on the Diagnostic Interview Schedule (DIS), Szadoczky and colleagues (2004) found low agreement between PCP and DIS diagnoses for anxiety and mood disorders [16].

This is in line with research outside of Hungary, where recognition rates of depression are under 10% in low and middle income countries [17], and approximately 50% in higher income countries [11]. Around half of PCPs recognize anxiety disorders [7], though recent literature assessing anxiety recognition in primary care is limited, and primarily focuses on generalized anxiety disorder (GAD). Less common disorders, such as obsessive-compulsive disorder (OCD), may be at an even greater risk for misdiagnosis. Studies of PCPs and general community members have found recognition rates as low as 15% [10, 18–20]. Lesser-known OCD presentations, such as obsessions related to harm or aggression or sexuality, tend to have the lowest recognition rates [10, 19, 20].

Demographic characteristics have been found to be predictive of whether individuals accurately identify mental health conditions, though no study has examined this in Hungary. Some research suggests that females are better at recognizing certain mental health conditions relative to males [18, 21, 22]. However, research on PCPs have not found a difference in recognition of mental health conditions based on gender, though the literature is sparse [23–25]. Research also suggests that older PCPs are less likely to recognize common mental health problems relative to younger PCPs [23, 25]. Moreover, municipality type (e.g., city vs. village) may also impact disorder recognition and treatment referral patterns. PCPs tend to prescribe more psychiatric medication in rural areas, where access to behavioral specialists is more sparse [26]. Rural residents also face additional disparities and are often at a disproportionately higher risk for mental health problems [27, 28]. As such, PCPs in rural areas may have greater knowledge of mental health conditions, as they may be tasked with seeing more mental health cases. Alternatively, as PCPs in rural areas may have fewer resources (e.g., mental health training workshops hosted in big cities), it is possible that mental health literacy may be poorer in these communities.

In addition to demographics, mental health stigma—or negative attitudes or stereotypes about people with mental illness—may also impact the identification of mental health conditions [29, 30]. Broadly, knowledge of mental disorders is associated with lower levels of mental health stigma [31]. Accordingly, mental health stigma may have a more detrimental impact on disorder recognition for lesser known or more stereotyped conditions. For example, OCD is often underrecognized in community and primary care settings (e.g [10, 20]), particularly when assessing more stigmatized symptoms (e.g., harm obsessions) [19, 20, 32]. Though often underrecognized, more stigmatized OCD symptoms tend to be more common in the general population than less stigmatized symptoms, such as contamination symptoms [33]. McCarthy and colleagues (2017) found an inverse relationship between OCD recognition and stigmatizing attitudes, such that those with greater stigmatizing attitudes recognized OCD less often, with more stigmatized symptoms recognized as OCD significantly less often than less stigmatized symptoms [19].

Furthermore, exposure to mental health, either via experiencing a mental health condition oneself or having a close family member/friend with a mental health condition, may also impact disorder recognition. A study of undergraduate students found that having a friend with a mental health condition increased the likelihood of correctly identifying social anxiety disorder (SAD) and GAD, though having a mental health condition oneself or having family member with a mental health condition was not associated with recognition [18]. In Hungary, psychiatrists who have sought help for their own mental health were more likely to have less social distance with their patients [34]. This in turn may be associated with improved disorder recognition, as some research has found an association between social distance and accurate disorder recognition [19, 31], though no study has examined this among Hungarian providers.

Misidentification of mental health conditions can also impact treatment recommendations and referrals [10, 35]. In a study examining US PCPs ability to recognize different presentations of OCD, PCPs who misdiagnosed OCD were less likely to recommend first-line treatment recommendations [10]. Inadequate treatment for anxiety and mood disorders are also generally more pronounced in primary care [35–37], with up to 79% of anxiety and mood disorder cases treated inadequately in primary care settings [35–39].

In summary, the ability of PCPs to appropriately identify and provide treatment recommendations and referrals for mental health conditions can have a substantial impact on both individual and societal functioning. Identifying knowledge gaps and characteristics (e.g., demographics) associated with misidentifying mental health conditions can inform PCP training and improve these key mental health outcomes. Therefore, the present study aimed to examine mental health literacy among PCPs in Hungary. Specifically, we aimed to examine how well PCPs recognize and provide appropriate treatment recommendations and referrals for common mental health concerns, namely, OCD harm/aggression subtype (OCD-Aggression), OCD order/symmetry subtype (OCD-Order), SAD, GAD, panic disorder (PD), and major depressive disorder (MDD). Our second aim was to examine the degree to which relevant demographic characteristics, including age, gender, and municipality, exposure to mental health (i.e., personal experiences and having a family member/friend with a mental health condition), and stigmatizing attitudes predict accurate identification of OCD, SAD, GAD, PD, and MDD.

## Method

### Participants

Participants were 208 PCPs aged 31 to 83 years (*M* = 55.89, *SD* = 10.84) recruited from the National Healthcare Fund (NEAK) in Hungary. See Table 1 for sample demographics.

**Table 1:** Hungarian primary care providers sample characteristics

Characteristic	*N*	%
Age (M years, +/- SD)	55.9	10.84
Years as PCP (M years, +/- SD)	20.7	12.88
Gender		
Male	79	38.0%
Female	129	62.0%
Patients Worked With		
Adults	117	56.3%
Children/Adolescents	52	25.0%
Both	39	18.8%
Received Treatment During Lifetime		
Yes	60	28.8%
No	145	69.7%
Rather not answer	3	1.4%
Diagnosed with Mental Illness		
Yes	15	7.2%
No	192	92.3%
Rather not answer	1	0.5%
Friend or Family with Mental Illness		
Yes	110	52.9%
No	96	46.2%
Rather not answer	2	1.0%
Municipality		
Capital (Budapest)	51	24.5%
County seat, city with county rights	47	22.6%
Other City	68	32.7%
Village	42	20.2%
County		
Baranya	7	3.4%
Heves	7	3.4%
Jász-Nagykun-Szolnok	7	3.4%
Komárom-Esztergom	3	1.4%
Nógrád	6	2.9%
Pest	34	16.3%
Somogy	3	1.4%
Szabolcs-Szatmár-Bereg	10	4.8%
Tolna	4	1.9%
Vas	8	3.8%
Veszprém	6	2.9%
Bács-Kiskun	9	4.3%
Zala	4	1.9%
Békés	8	3.8%
Borsod-Abaúj-Zemplé	14	6.7%
Budapest	39	18.8%
Csongrád-Csanád	5	2.4%
Fejér	12	5.8%
Győr-Moson-Sopron	12	5.8%
Hajdú-Bihar	10	4.8%

Note. Data is presented for the entire sample, *n* = 208

### Procedures

Surveys were sent to 6,432 PCPs in the NEAK database in December 2023. Participants received a reminder email approximately 3 weeks later, in January 2024. The questionnaire was completed online using a link hosted by the National Directorate General for Hospitals (Országos Kórházi Főigazgatóság) internal server from December 2022 to January 2023. Interested participants were provided with an informed consent page upon initiating the survey and indicated whether they agreed to participate in the study by checking a yes or no box. Of the 211 participants who initiated the survey, all participants consented to participate. Participants were excluded if they indicated that they were not a currently licensed and practicing general practitioner (*n* = 3). The final sample resulted in 208 PCPs. The local National Scientific and Ethical Committee approved all study procedures (TUKEB # BM/30518-1/2023).

The online survey assessed demographics, exposure to mental health (i.e., personal experience and having a family member/friend with a mental health condition), and stigmatizing attitudes towards people with mental illness. Participants also read six vignettes describing an individual experiencing OCD with symptoms related to harm or aggression (i.e., OCD-Aggression), OCD with symptoms of order/symmetry (i.e., OCD-Order), GAD, SAD, PD, and MDD (see Materials/Measures). After reading each vignette, participants answered questions assessing disorder recognition, perceived cause of the disorder, and treatment recommendations and referrals. The age of the person described in each vignette was adapted based on whether the participant indicated that they primarily work with children/adolescents, adults, or both. For those who indicated both, three vignettes were described a child/adolescent, and three vignettes described an adult. All participants responded to all six vignettes, however, vignettes were counterbalanced, such that participants received vignettes in a randomized order to reduce order effects.

### Materials/Measures

#### Demographics and personal experiences with mental health

Participants completed a questionnaire assessing age, sex, municipality size (e.g., capital (Budapest), big city (capital of the county), medium-sized city (> 50,000 inhabitants), small town (< 50,000 inhabitants/village)), county of residence, and experience as a PCPs (i.e., number of years practicing, specialty area). Personal experience with mental illness was assessed through a yes/no response to the question, “Have you ever been diagnosed with a mental illness?” and “In your lifetime, have you received any treatment for emotional or mental health problems (e.g., therapy, counseling, medication)?” To assess exposure to people with a mental health condition, participants were asked “Do you have a friend or family member who is experiencing mental illness?”

#### Opening minds stigma scale for health care providers (OMS-HC)

The OMS-HC is a 15-item self-report questionnaire that measures stigmatizing attitudes towards individuals with mental health problems in healthcare providers [40, 41]. The OMS-HC consists of three subscales—Attitude, Disclosure and Help-seeking, and Social Distance—which can be summed to create a total score representing participant’s overall attitude/stigma towards mental health. Items are rated on a 5-point Likert Scale from 1 (strongly agree) to 5 (strongly disagree), with total scores range from 15 to 75 and higher scores indicating greater stigma towards mental health. The OMS-HC has been translated and validated in Hungarian and demonstrates good to excellent reliability and validity [42]. In the Hungarian version of the OMS-HC, item 11 did not load strongly enough on any of the three factors, therefore, it is recommended to omit this item from the total score [42]. Therefore, total OMS-HC scores used in the present study are based on the 14 item OMS-HC, with total scores ranging from 14 to 70.

#### Vignettes

Six vignettes were adapted from prior studies (see Appendix A), with permission obtained from the first authors [18, 20]. Vignettes followed recommendations for appropriate length and reading comprehension and were designed to portray disorder symptoms based on DSM criteria and following prior published studies [18, 20]. Two vignettes described individuals experiencing symptoms of OCD—one depicting symptoms of aggression (e.g., OCD-Aggression; obsessions and compulsions related to harm) and the other depicting symptoms of order/symmetry (e.g., OCD-Order; obsessions and compulsions related to rearranging items and having everything be “just right”), adapted from García-Soriano and Roncero (2017). The other four vignettes depicted symptoms of GAD, SAD, PD, and MDD, adapted from Coles and Coleman (2010). The original vignettes were created in English. Vignettes were translated into Hungarian by a bilingual professional in psychiatry. Vignettes were then back translated by another professional in psychiatry. The back translated version of the vignettes were then compared with the original English version by a third member of the research team and discrepancies were discussed among the group of three translators. A sample of 5 individuals then read vignettes for concept checking. See Appendix B for Hungarian translations of each vignette.

#### Vignette response questions

After reading each vignette, participants were provided with an open-ended response to the question, “Is something wrong with the person described in the vignette? That is, what do you think they would be diagnosed with?” Following prior published work on open-ended labels [19], any response containing part or all of the condition described (e.g., “OCD” or “obsessive-compulsive”) was coded as a correct response. Responses in which the participant listed more than one disorder (e.g., autism and OCD) were also coded as a correct response as long as one of the disorders listed was correct. Participants subsequently answered three additional questions assessing perceived cause of the condition, whether or not they believe the person described in the vignette needs help, and ranked their top three treatment referrals/recommendations. Participants who endorsed “medication” as one of their top three treatment recommendations were asked to provide an open-ended response to the medication they would recommend. Open-ended medication responses were coded into seven categories: Anxiolytic (e.g., Benzodiazepines); Selective Serotonin Reuptake Inhibitors (SSRIs; e.g., Escitalopram); Antipsychotic (e.g., Haloperidol); Tricyclics (e.g., Clomipramine); More than one medication recommended; Uncertain/Unknown; and Other. See Appendix C for all question items.

### Data analysis

Analyses were performed using SPSS 29.0. Descriptive analyses were used to characterize disorder recognition rates, perceived disorder causes, and treatment recommendations. Six binary logistic regression analyses were conducted to examine the degree to which demographic characteristics (age, sex, municipality), mental health stigma (OMS-HC total score), and exposure to mental health (personal experiences and having a family member/friend with a mental health condition) predict ability to correctly characterize each vignette. Predictors were selected a priori based on prior literature. As a Pearson correlation analysis indicated a large correlation (*r* = .683, *p* < .001) between age and number of years practicing, we opted to only include age as a predictor in the regression models. Age and OMS-HC total scores were entered as continuous variables. Gender, municipality, and exposure to mental health were entered as categorical variables.

## Results

### Descriptive analyses

Sample characteristics are reported in Table 1. The majority of the sample were female (*n* = 129; 62.0%). On average, participants had been working as a PCP for 20.07 years (*SD* = 12.88 years). Over half (*n* = 117; 56.3%) of PCPs endorsed working primarily with adults, 25.0% (*n* = 52) endorsed working primarily with children/adolescents, and 18.8% (*n* = 39) endorsed working with both children/adolescents and adults. Approximately one-fourth (*n* = 60; 28.8%) of participants endorsed receiving mental health treatment in their lifetime. Only fifteen participants (7.2%) endorsed ever being diagnosed with a mental health problem. More than half (*n* = 110; 52.9%) of participants endorsed having a close family member/friend experiencing a mental health condition. The average OMS-HC score among participants was 32.09 (*SD* = 7.45).

### Disorder recognition rates

Rates of recognition and treatment recommendations and referrals for each disorder are summarized in Tables 2, 3, 4, 5, 6 and 7. For OCD-Aggression, 23.9% (*n* = 58) of the participants correctly labeled the vignette as OCD, with the highest number of participants (*n* = 67; 32.2%) labeling the vignette as Schizophrenia. For OCD-Order, 75.5% (*n* = 157) of participants correctly labeled the vignette as OCD. A minority of participants (*n* = 71; 34.1%) correctly recognized SAD, with anxiety as the most common alternative response (*n* = 68; 32.7%). The majority of participants correctly identified GAD (*n* = 158; 76.0%), PD (*n* = 164; 78.8%), and MDD (*n* = 190; 91.3%).

**Table 2:** Vignette 1: Obsessive – Compulsive Disorder – Aggression

Label Assigned (Open-Ended)	*N*	% of total sample (*n* = 208)
Obsessive-Compulsive Disorder	58	27.9%
**Schizophrenia**	**67**	**32.2%**
Depression/Suicidality	20	9.6%
Personality Disorder	11	5.3%
Other (e.g., panic disorder, adjustment disorder)	24	11.5%
Listed more than one incorrect diagnosis (e.g., bipolar and schizophrenia)	8	3.8%
Non-specific response^1^	20	9.6%
Medication Recommendation (Open-Ended)	*N*	% endorsing medication (*n* = 112)*
Anti-Anxiety	15	13.4%
Selective Serotonin Reuptake Inhibitors (SSRIs)	21	18.7%
**Antipsychotics**	**47**	**42.0%**
Tricyclics	1	0.9%
More than one medication recommended	6	5.4%
Uncertain/Unknown	21	18.7%
Other	1	0.9%
First Choice Treatment Recommendation (Multiple-Choice)	*N*	% of total sample (*n* = 208)
Therapist or Psychologist	30	14.4%
**Psychiatrist**	**131**	**63.0%**
Medication	4	1.9%
General Practitioner	12	5.8%
Social Support (friend, family, teacher)	4	1.9%
Hospital/Emergency Care	25	12.0%
Priest or Church	2	1.0%
Concealment	0	0.0%
Other	0	0.0%
Primary Cause (Multiple-Choice)	*N*	%% of total sample (*n* = 208)
**Mental Illness**	**190**	**91.3%**
Stress	6	2.9%
Biological Factors	6	2.9%
Environmental Factors	1	0.5%
Personal Weakness	2	1.0%
Other	3	1.5%

Note. *Only participants who endorsed medication as one of their top three treatment recommendations were asked what medication they would recommend; ^1^Participants were coded as having a non-specific response if they indicated that there was a problem but did not specify the type of problem. For example, several participants indicated “Yes, this person has a problem.” Data is presented for all available cases, *n* = 208. The most common responses are bolded

**Table 3:** Vignette 2: Obsessive compulsive disorder – order/symmetry symptoms

Label Assigned (Open-Ended)	*N*	% of total sample (*n* = 208)
**Obsessive Compulsive Disorder**	**157**	**75.5%**
Autism	6	2.9%
Bipolar Disorder	6	2.9%
Anxiety	5	2.4%
Obsessive Compulsive Personality Disorder	5	2.4%
Other (e.g., phobia)	15	7.2%
Listed more than one incorrect diagnosis (e.g., manic and schizoid)	6	2.9%
Non-specific response^1^	8	3.8%
Medication Recommendation (Open-Ended)	*N*	% endorsing medication (*n* = 95)*
Anxiolytics	17	17.9%
**Selective Serotonin Reuptake Inhibitors (SSRIs)**	**38**	**40.0%**
Antipsychotics	6	6.3%
Tricyclics	3	3.2%
More than one medication recommended	13	13.7%
Uncertain/Unknown	13	13.7%
Other	5	5.3%
First Choice Treatment Recommendation (Multiple-Choice)	*N*	% of total sample (*n* = 208)
Therapist or Psychologist	72	34.6%
**Psychiatrist**	**112**	**53.8%**
Medication	4	1.9%
General Practitioner	15	7.2%
Social Support (friend, family, teacher)	2	1.0%
Hospital/Emergency Care	2	1.0%
Priest or Church	1	0.5%
Concealment	0	0.0%
Other	0	0.0%
Primary Cause (Multiple-Choice)	*N*	% of total sample (*n* = 208)
**Mental Illness**	**185**	**86.9%**
Stress	12	5.8%
Biological Factors	5	2.4%
Environmental Factors	1	0.5%
Personal Weakness	4	1.9%
Other	1	0.5%

Note. *Only participants who endorsed medication as one of their top three treatment recommendations were asked what medication they would recommend; ^1^Participants were coded as having a non-specific response if they indicated that there was a problem but did not specify the type of problem. For example, several participants indicated “Yes, this person has a problem.” Data is presented for all available cases, *n* = 208. The most common responses are bolded

**Table 4:** Vignette 3: Social anxiety disorder vignette

Label Assigned (Open-Ended)	*N*	% of total sample (*n* = 208)
**Social Anxiety Disorder**	**71**	**34.1%**
Anxiety	68	32.7%
Personality flaw (e.g., lack of self-confidence)	12	5.8%
Personality Disorder	5	2.4%
Adjustment Disorder	4	1.9%
Other (e.g., autism, panic)	23	11.1%
Listed more than one incorrect diagnosis (e.g., OCD and depression)	16	7.7%
Non-specific response^1^	9	4.3%
Medication Recommendation (Open-Ended)	*N*	% endorsing medication (*n* = 49)*
**Anxiolytics**	**30**	**61.2%**
Selective Serotonin Reuptake Inhibitors (SSRIs)	9	18.4%
More than one medication recommended	7	14.3%
Uncertain/Unknown	1	2.0%
Other	2	4.1%
First Choice Treatment Recommendation (Multiple-Choice)	*N*	% of total sample (*n* = 208)
**Therapist or Psychologist**	**122**	**58.7%**
Psychiatrist	20	9.6%
Medication	1	0.5%
General Practitioner	21	10.1%
Social Support (friend, family, teacher)	43	20.7%
Hospital / Emergency room	0	0.0%
Priest or Church	0	0.0%
Concealment	0	0.0%
Other	1	0.5%
Primary Cause (Multiple-Choice)	*N*	% of total sample (*n* = 208)
**Mental Illness**	**102**	**49.0%**
Stress	42	20.2%
Biological Factors	11	5.3%
Environmental Factors	18	8.7%
Personal Weakness	28	13.5%
Other	7	3.5%

Note. *Only participants who endorsed medication as one of their top three treatment recommendations were asked what medication they would recommend; ^1^Participants were coded as having a non-specific response if they indicated that there was a problem but did not specify the type of problem. For example, several participants indicated “Yes, this person has a problem.”; Data is presented for all available cases, *n* = 208. The most common responses are bolded

**Table 5:** Vignette 4: Generalized anxiety disorder vignette

Label Assigned (Open-Ended)	*N*	% of total sample (*n* = 208)
**Generalized Anxiety Disorder**	**158**	**76.0%**
Stress/Tension	8	3.8%
Obsessive-Compulsive Disorder	6	2.9%
Other (e.g. medical problem, personality issue)	23	11.0%
Listed more than one incorrect diagnosis (e.g., “phobia, paranoia”)	3	1.4%
Non-specific response^1^	10	4.8%
Medication Recommendation (Open-Ended)	*N*	% endorsing medication (*n* = 72)*
**Anxiolytics**	**32**	**44.4%**
Selective Serotonin Reuptake Inhibitors (SSRIs)	16	22.2%
Tricyclics	1	1.4%
More than one medication recommended	15	20.8%
Uncertain/Unknown	3	4.2%
Other	5	6.9%
First Choice Treatment Recommendation (Multiple-Choice)	*N*	% of total sample (*n* = 208)
**Therapist or Psychologist**	**101**	**48.6%**
Psychiatrist	26	12.5%
Medication	4	1.9%
General Practitioner	34	16.3%
Social Support (friend, family, teacher)	39	18.8%
Hospital / Emergency Room	0	0.0%
Priest or Church	3	1.4%
Concealment	0	0.0%
Other	1	0.5%
Primary Cause (Multiple-Choice)	*N*	% of total sample (*n* = 208)
**Mental Illness**	**83**	**39.9%**
Stress	77	37.0%
Biological Factors	11	5.3%
Environmental Factors	16	7.7%
Personal Weakness	11	5.3%
Other	10	5.0%

Note. *Only participants who endorsed medication as one of their top three treatment recommendations were asked what medication they would recommend; ^1^Participants were coded as having a non-specific response if they indicated that there was a problem but did not specify the type of problem. For example, several participants indicated “Yes, this person has a problem.”; Data is presented for all available cases, *n* = 208. The most common responses are bolded

**Table 6:** Vignette 6: Panic disorder vignette

Label Assigned (Open-Ended)	*N*	% of total sample (*n* = 208)
**Panic Disorder**	**164**	**78.8%**
Medical Problem	18	8.7%
Anxiety	7	3.4%
Other (e.g., stress, tension)	10	4.8%
Listed more than one incorrect diagnosis (e.g., “phobia, paranoia”)	2	1.0%
Non-specific response^1^	7	3.4%
Medication Recommendation (Open-Ended)	*N*	% endorsing medication (*n* = 90)*
**Anxiolytics**	**42**	**46.7%**
Selective Serotonin Reuptake Inhibitors (SSRIs)	26	28.9%
More than one medication recommended	14	15.6%
Uncertain/Unknown	5	5.6%
Other	3	3.3%
First Choice Treatment Recommendation (Multiple-Choice)	*N*	% of total sample (*n* = 208)
Therapist or Psychologist	68	32.7%
Psychiatrist	31	14.9%
Medication	5	2.4%
**General Practitioner**	**83**	**39.9%**
Social Support (friend, family, teacher)	8	3.8%
Hospital/Emergency Care	8	3.8%
Priest or Church	0	0.0%
Concealment	0	0.0%
Other	5	2.4%
Primary Cause (Multiple-Choice)	*N*	% of total sample (*n* = 208)
**Mental Illness**	**107**	**51.4%**
Stress	61	29.3%
Biological Factors	22	10.6%
Environmental Factors	8	3.8%
Personal Weakness	5	2.4%
Other	5	2.5%

Note. *Only participants who endorsed medication as one of their top three treatment recommendations were asked what medication they would recommend; ^1^Participants were coded as having a non-specific response if they indicated that there was a problem but did not specify the type of problem. For example, several participants indicated “Yes, this person has a problem.”; Data is presented for all available cases, *n* = 208. The most common responses are bolded

**Table 7:** Vignette 7: Major depressive disorder vignette

Label Assigned (Open-Ended)	*N*	% of total sample (*n* = 208)
**Depression**	**190**	**91.3%**
Anxiety	2	1.0%
Other (e.g. medical problem)	9	4.3%
Non-specific response^1^	7	3.4%
Medication Recommendation (Open-Ended)	*N*	% endorsing medication (*n* = 114)*
Anxiolytics	3	2.6%
**Selective Serotonin Reuptake Inhibitors (SSRIs)**	**95**	**83.3%**
Tricyclics	1	0.9%
More than one medication recommended	11	9.6%
Uncertain/Unknown	1	0.9%
Other	3	2.6%
First Choice Treatment Recommendation (Multiple-Choice)	*N*	% of total sample (*n* = 208)
Therapist or Psychologist	36	17.3%
**Psychiatrist**	**97**	**46.6%**
Medication	12	5.8%
General Practitioner	45	21.6%
Social Support (friend, family, teacher)	15	7.2%
Hospital/Emergency Care	2	1.0%
Priest or Church	0	0.0%
Concealment	0	0.0%
Other	1	0.5%
Primary Cause (Multiple-Choice)	*N*	% of total sample (*n* = 208)
**Mental Illness**	**157**	**75.5%**
Stress	17	8.2%
Biological Factors	14	6.7%
Environmental Factors	14	6.7%
Personal Weaknesss	3	1.4%
Other	3	1.5%

Note. *Only participants who endorsed medication as one of their top three treatment recommendations were asked what medication they would recommend; ^1^Participants were coded as having a non-specific response if they indicated that there was a problem but did not specify the type of problem. For example, several participants indicated “Yes, this person has a problem.”; Data is presented for all available cases, *n* = 208. The most common responses are bolded

### Disorder causes

The majority of participants attributed mental illness to be the primary cause for the OCD-Aggression (*n* = 190; 91.3%), OCD-Order (*n* = 185; 88.9%), and MDD (*n* = 157; 75.5%) vignettes. Few participants (0.5–8.2%) endorsed stress, biological factors, environmental factors, or personal weakness as potential causes for these conditions (see Tables 2 and 3, and 7). For SAD (Table 4), while 49.0% (*n* = 102) attributed the problem to mental illness, other common responses were stress (*n* = 42; 20.2%), personal weakness (*n* = 28; 13.5%), and environmental factors (*n* = 18; 8.7%), with 11 participants (5.3%) endorsing biological factors. For GAD (Table 5), mental illness (*n* = 83; 39.9%), stress (*n* = 77; 37.0%), and environmental factors (*n* = 16; 7.7%) were the most common responses, with few participants endorsing biological factors (*n* = 11; 5.3%) and personal weakness (*n* = 11; 5.3%). Finally, the most commonly selected primary causes for PD (Table 6) were mental illness (*n* = 107; 51.4%), stress (*n* = 61, 29.3%), and biological factors (*n* = 22, 10.6%). Environmental factors (*n* = 8; 3.8%) and personal weakness (*n* = 5; 2.4%) were not common responses for PD.

### Treatment recommendations

A psychiatrist was the most common first-choice treatment referral for OCD-Aggression (*n* = 131; 63.0%;), OCD-Order (*n* = 112; 53.8%), and MDD (*n* = 97; 46.6%). Seeing a therapist/psychologist was the most common first-choice treatment referral for SAD (*n* = 122; 58.7%) and GAD (*n* = 101; 48.6%); Seeing a general practitioner was the most common for PD (*n* = 83; 39.9%). Regarding medication recommendations, out of participants who endorsed medication as a top three treatment recommendation for OCD-Aggression (*n* = 112; 53.8%), the most common recommendation was anti-psychotics (*n* = 47; 42.0%). Of those who endorsed medication as a treatment recommendation for OCD-Order (*n* = 95; 45.7%) and MDD (*n* = 114; 54.8%), SSRIs were the most common recommendation (OCD: *n* = 38; 40.0%; MDD: *n* = 95; 83.3%). Of those who endorsed medication as a treatment recommendation for SAD (*n* = 49; 23.6%), GAD (*n* = 72; 34.6%), and PD (*n* = 90; 43.3%), anxiolytics were the most common recommendation (SAD: *n* = 30; 61.2%; GAD: *n* = 32; 44.4%; PD: *n* = 42; 46.7%). See Tables 2, 3, 4, 5, 6 and 7 for all descriptive statistics.

### Predictors of disorder recognition

Results from the six binary logistic regressions are reported in Table 8. Older PCPs were more likely to mislabel OCD-Aggression (OR: 1.04; Wald *χ*^2^(1) = 4.70, *p* = .030), PD (OR: 1.04; Wald *χ*^2^(1) = 5.23, *p* = .022) and MDD (OR: 1.09; Wald *χ*^2^(1) = 6.94, *p* = .008) relative to younger PCPs. Male PCPs were significantly more likely to mislabel GAD than female PCPs (OR: 0.339; Wald *χ*^2^(1) = 8.79, *p* = .003). PCPs with greater reported mental health stigma were significantly more likely to mislabel OCD-Order relative to PCPs with lower stigma (OR: 0.950; Wald *χ*^2^(1) = 4.36, *p* = .037). Finally, PCPs who reported not having a family member/friend with a mental health condition were significantly more likely to mislabel SAD (OR: 2.01; Wald *χ*^2^(1) = 4.12, *p* = .042).

**Table 8:** Predictors of disorder recognition

		Ꞵ	SE	Wald	*p*		95% CI for OR		
Disorder						OR	Lower	Upper	
OCD-Agg	Constant	-2.80	1.62	2.97	0.085	0.61			
	**Age**	**0.04**	**0.019**	**4.70**	**0.030**	**1.04**	**1.00**		**1.08**
	Sex (Reference = Male)								
	Female	-0.27	0.39	0.48	0.488	0.77	0.36		1.63
	Municipality (Reference = Budapest)			1.04	0.791				
	County seat, city w/ county rights	-0.28	0.57	0.24	0.627	0.76	0.25		2.33
	Other City	0.27	0.47	0.32	0.572	1.30	0.52		3.27
	Village	0.12	0.54	0.05	0.822	1.13	0.39		3.26
	Received Treatment (Reference = Yes)								
	No	-0.12	0.48	0.00	0.980	0.99	0.39		2.53
	Received Diagnosis (Reference = Yes)								
	No	0.51	0.78	0.43	0.511	1.67	0.36		7.65
	Family/Friend with Mental Illness (Reference = Yes)								
	No	0.50	0.40	1.58	0.209	1.65	0.76		3.59
	OMS-HC Total Score	0.01	0.03	0.15	0.696	1.01	0.96		1.06
OCD-Order	Constant	-1.17	1.50	0.60	0.437	0.31			
	Age	0.03	0.18	3.06	0.080	1.03	1.00		1.07
	Sex (Reference = Male)								
	Female	0.07	0.38	0.04	0.849	1.07	0.51		2.25
	Municipality (Reference = Budapest)			0.62	0.892				
	County seat, city w/ county rights	-0.33	0.55	0.36	0.547	0.72	0.25		2.10
	Other City	0.02	0.53	0.00	0.964	1.02	0.36		2.88
	Village	-0.21	0.50	0.18	0.675	0.81	0.31		2.15
	Received Treatment (Reference = Yes)								
	No	0.36	0.49	0.54	0.465	1.43	0.55		3.72
	Received Diagnosis (Reference = Yes)								
	No	-0.78	0.77	1.02	0.312	0.46	0.10		2.07
	Family/Friend with Mental Illness (Reference = Yes)								
	No	0.67	0.38	3.06	0.080	1.95	0.92		4.13
	**OMS-HC Total Score**	**-0.05**	**0.03**	**4.36**	**0.037**	**0.95**	**0.91**		**1.00**
GAD	Constant	-3.59	1.77	4.14	0.042	0.28			
	Age	0.02	0.02	1.65	0.200	1.02	0.99		1.06
	**Sex (Reference = Male)**								
	**Female**	**-1.08**	**0.37**	**8.79**	**0.003**	**0.34**	**0.17**		**0.69**
	Municipality (Reference = Budapest)			3.92	0.271				
	County seat, city w/ county rights	-0.25	0.54	0.22	0.638	0.78	0.27		2.23
	Other City	-0.77	0.60	1.67	0.196	0.46	0.14		1.49
	Village	0.25	0.48	0.26	0.609	1.28	0.50		3.31
	Received Treatment (Reference = Yes)								
	No	0.18	0.47	0.15	0.703	1.20	0.48		3.02
	Received Diagnosis (Reference = Yes)								
	No	1.14	1.15	0.98	0.322	3.13	0.33		29.93
	Family/Friend with Mental Illness (Reference = Yes)								
	No	0.06	0.37	0.02	0.883	1.06	0.51		2.19
	OMS-HC Total Score	0.02	0.02	0.54	0.465	1.02	0.97		1.07
SAD	Constant	1.66	1.44	1.33	0.249	5.28			
	Age	0.00	0.02	0.00	0.997	1.00	0.97		1.03
	Sex (Reference = Male)								
	Female	-0.66	0.35	3.54	0.060	0.52	0.26		1.03
	Municipality (Reference = Budapest)			2.11	0.550				
	County seat, city w/ county rights	-0.22	0.48	0.21	0.646	0.80	0.31		2.06
	Other City	0.18	0.51	0.13	0.719	1.20	0.44		3.28
	Village	0.37	0.48	0.61	0.435	1.45	0.57		3.70
	Received Treatment (Reference = Yes)								
	No	0.21	0.42	0.26	0.614	1.23	0.55		2.78
	Received Diagnosis (Reference = Yes)								
	No	-0.37	0.68	0.31	0.580	0.69	0.18		2.59
	**Family/Friend with Mental Illness (Reference = Yes)**								
	**No**	**0.70**	**0.35**	**4.12**	**0.042**	**2.01**	**1.03**		**3.96**
	OMS-HC Total Score	-0.03	0.02	1.49	0.222	0.97	0.93		1.02
PD	Constant	-3.72	1.64	5.16	0.023	0.02			
	**Age**	**0.04**	**0.02**	**5.23**	**0.022**	**1.04**	**1.01**		**1.08**
	Sex (Reference = Male)								
	Female	-0.51	0.37	1.94	0.164	0.60	0.29		1.23
	Municipality (Reference = Budapest)			2.66	0.446				
	County seat, city w/ county rights	0.77	0.53	2.12	0.146	2.16	0.77		6.11
	Other City	0.21	0.51	0.16	0.685	1.23	0.46		3.31
	Village	0.14	0.58	0.06	0.804	1.15	0.37		3.58
	Received Treatment (Reference = Yes)								
	No	-0.25	0.47	0.29	0.593	0.78	0.31		1.95
	Received Diagnosis (Reference = Yes)								
	No	-0.14	0.82	0.03	0.868	0.87	0.18		4.32
	Family/Friend with Mental Illness (Reference = Yes)								
	No	0.71	0.38	3.47	0.063	2.04	0.96		4.31
	OMS-HC Total Score	0.00	0.02	0.00	0.977	1.00	0.96		1.05
MDD	Constant	-5.24	2.57	4.14	0.042	0.01			
	**Age**	**0.08**	**0.03**	**6.94**	**0.008**	**1.09**	**1.02**		**1.16**
	Sex (Reference = Male)								
	Female	-1.05	0.58	3.26	0.071	0.35	0.11		1.09
	Municipality (Reference = Budapest)			0.13	0.988				
	County seat, city w/ county rights	0.09	0.92	0.01	0.926	1.09	0.18		6.60
	Other City	0.29	0.87	0.11	0.735	1.34	0.24		7.40
	Village	0.09	0.80	0.01	0.908	1.10	0.23		5.25
	Received Treatment (Reference = Yes)								
	No	0.10	0.85	0.01	0.908	1.10	0.21		5.87
	Received Diagnosis (Reference = Yes)								
	No	-0.35	1.37	0.06	0.800	0.71	0.05		10.39
	Family/Friend with Mental Illness (Reference = Yes)								
	No	0.68	0.61	1.25	0.264	1.98	0.60		6.53
	OMS-HC Total Score	-0.06	0.04	2.41	0.121	0.94	0.87		1.07

Note. B = ordered log odds coefficient; SE = standard error; OR = Odds Ratio; OMS-HC = Opening Minds Stigma Scale for Health Care Providers; OCD-Aggression = Obsessive-Compulsive Disorder with aggression/harm symptoms; OCD-Order = Obsessive-Compulsive Disorder with order/symmetry symptoms; GAD = Generalized Anxiety Disorder; SAD = Social Anxiety Disorder; PD = Panic Disorder; MDD = Major Depressive Disorder. Significant findings (*p* < .05) are bolded

## Discussion

As primary care is often the first step in identifying mental health problems and receiving care, the ability of PCPs to recognize common mental health conditions is paramount. The present study examined how well PCPs in Hungary recognize and provide appropriate treatment referrals and recommendations for OCD-Aggression, OCD-Order, SAD, GAD, PD, and MDD. We also examined the degree to which demographic characteristics, mental health stigma, and exposure to mental health predict whether PCPs correctly recognized each condition. Overall, we found that Hungarian PCPs generally accurately label common mental health conditions (i.e., over 70% recognized GAD, PD, OCD-Order, and MDD), though low identification rates of OCD-Aggression (23.9%) and SAD (34.1%) are noteworthy. Similarly, treatment referrals were largely accurate (e.g., the majority of participants recommended a psychiatrist or psychologist/therapist), except for PD, wherein the most common treatment referral was a PCP. Medication recommendations warrant attention, as the majority of participants recommended anxiolytics as the first line recommendation for GAD, SAD, and PD, which are typically not considered a first-line intervention for anxiety disorders. Finally, our findings suggest that older physicians, men, stigma, and lack of exposure to mental health conditions personally (i.e., not having a family member/friend with a mental health condition) may be added barriers to the accurate identification of mental health conditions, suggesting potential targets for educational interventions.

OCD is a highly disabling and commonly misdiagnosed condition, having a profound and debilitating impact on people all across the world [43]. Misdiagnosis is common among both general community members, medical professionals, and mental health professionals. Lesser known symptom presentations, such as aggression (i.e., harm obsessions), may be most vulnerable to misdiagnosis [10]. Thus, not surprisingly, in the present study, OCD-Aggression was the most underrecognized condition, with only 23.9% of providers accurately labeling the vignette as OCD, and most commonly labeling it as schizophrenia (32.2%). This is consistent with prior research similarly finding OCD-Aggression to be recognized by only 20.0% of US PCPs [10] and 23.5% of Spanish adolescents [20], who similarly mislabeled it as schizophrenia [10, 20]. Both in the present study and prior literature, other symptom presentations of OCD have better recognition rates. OCD-Order was recognized by 75.5% of participants in the present study, aligning with prior research finding similarly high recognition rates for OCD when symptoms depicted order, symmetry, or contamination [18–20]. This is likely due to increased media coverage of contamination and order symptoms of OCD, relative to other OCD presentations [20, 44, 45]. Nonetheless, it highlights an important knowledge gap among PCPs in Hungary. While PCPs are not tasked with treating OCD, increasing awareness of OCD and its heterogenous presentations may help improve referrals and reduce mental health stigma in the broader community. Educational interventions aimed at improving knowledge and attitudes towards OCD have shown preliminary benefit [46], and may be worth considering in the Hungarian PCP community.

GAD, PD, OCD-Order, and MDD were all recognized by over 70% of the sample. These recognition rates exceed that of findings prior research on undergraduate students in the United States, who exhibited recognition rates of 47.7% and 41.4% for PD and GAD [18], respectively, though recognition rates for OCD-Order were slightly higher in prior research on Spanish adolescents (84.3%; [20]) and American adults (84.5%; [19]). MDD had the highest recognition rate, with 91.3% giving it a correct label. At first sight this is surprising, given that prior research on Hungarian PCPs found low agreement between DIS diagnosed mood disorders and PCP diagnosed mood disorders [16]. However, this high recognition rate could be attributed to the increase in Continuing Medical Education (CME) programs across Hungary in the last two decades. These trainings focused primarily on depression recognition, management, and suicide prevention, which could explain the high recognition rate of depression among our sample. In addition to CME training, two interventions targeting improving PCPs’ literacy of depression and suicide risk have occurred in Hungary in the last two decades (e.g [47–49]), which may have contributed to the improved recognition rate from past studies [16]. These findings may also be reflected in the markedly increased antidepressant use and steadily declining suicide rate of Hungary in the last two to three decades [50, 51]. Another possible explanation is that vignette-based disorder recognition is easier than detection in real time (e.g., comparing diagnoses given by PCPs to clinical interviews), as done in Szadoczky and colleagues (2004). Indeed, studies using vignettes in US undergraduate samples found that 88% of students correctly recognized depression [18]. Nonetheless, the present findings show strength in Hungarian PCP’s ability to recognize common mental health conditions, and suggest that similar interventions (e.g., CME training) for other conditions (e.g., SAD, OCD-Aggression, or those beyond the scope of this study) may further improve recognition rates.

Perceiving a mental health condition to be caused by mental illness (i.e., rather than stress or personal weakness) is associated with more appropriate treatment recommendations [18, 52]. In the present study, the most commonly selected perceived primary cause for each condition was mental illness. These findings are favorable compared to prior research in American undergraduate students, where the most common selected perceived cause were personal weakness, stress, and biological factors for SAD, PD, GAD, and MDD [18]. Nonetheless, in the present study, a considerable number of participants also selected stress as the primary cause for the anxiety disorders. This is not surprising, as some evidence suggest that PCPs consider mental health conditions a personal weakness, rather than mental illness [53]. Taken together, our findings suggest that Hungarian PCP’s may be more adept at recognizing causes for mental illness than found in prior work with other populations, though further emphasis on the causes of anxiety disorders should be considered.

Treatment referrals varied based on condition. Seeing a psychiatrist was the most common first choice referral for OCD-Aggression, OCD-Order, and MDD. Seeing a therapist or psychologist was the most common first choice referral for SAD and GAD. Surprisingly, seeing a PCP was the most common first choice treatment referral for PD (39.9%). This may be because symptoms of PD often present similarly to other physical health conditions and many people experiencing PD resort to the emergency room when experiencing a panic attack [54, 55]. Thus, while PD was commonly recognized in the present study, our data suggest that PCPs are less clear on which type of provider would be the most efficacious for treating PD.

Anxiolytics (e.g., benzodiazepines) were the most commonly recommended medication for the anxiety disorders. Current treatment guidelines for anxiety disorders do not endorse the use of anxiolytics in the long term, and recommend SSRIs are the first line intervention [56, 57]. However, the use of anxiolytics remains widespread, and some researchers suggest that it may be as efficacious and safe as SSRIs [58]. Nonetheless, anxiolytics are associated with abuse, tolerance, and other serious side effects, particularly in cases of inadequate indication, prolonged administration, inappropriate doses, and in those with comorbid substance use disorders [59, 60]. Notably, research suggest that the majority of anxiolytics are prescribed by PCPs, and that there have been an increase in PCP prescriptions over time [61–63]. Another study found that PCPs were more likely to prescribe benzodiazepines to patients more vulnerable to benzodiazepine-related adverse events (e.g., older patients) [62]. Taken together, these findings highlight a need for increased psychoeducation among PCPs on the appropriate use of anxiolytic medication.

Binary logistic regression analyses revealed that older PCPs were more likely to mislabel OCD-Aggression, PD, and MDD relative to younger PCPs. This is consistent with research finding lower recognition rates of common mental health problems among older physicians [23, 25], and that psychiatric patients experienced poorer outcomes when treated by an older physician, though this study notes that this could be due to the fact that older psychiatrists may be assigned to more severe patients [64]. Additionally, male PCPs were more likely to mislabel GAD than female PCPs. Prior research on recognition of common mental health conditions based on clinician gender have mixed results, with some finding no effect of gender [23–25]. However, medical research has found that female physicians are more likely to adhere to evidence-based practice and guidelines [65] and provide more patient-focused communication and psychosocial support [66, 67], potentially lending itself to improved mental health condition recognition. Another possible explanation for gender-based recognition differences may be due to personal treatment utilization. A network analysis of Hungarian community members revealed that females were more likely than males to have received mental health treatment in their lifetime, irrespective of clinical symptoms, and this association bridged the relationship between being female and having more positive attitudes toward mental health help-seeking [68].

PCPs with greater reported mental health stigma were significantly more likely to mislabel OCD-Order relative to PCPs with lower stigma. This is surprising, as research examining stigma and OCD symptom presentations have found OCD-Order presentations to be less stigmatizing relative to other symptom presentations, such as harm obsessions (i.e., OCD-Aggression) [19, 69]. One possible explanation is related to our assessment of mental health stigma. In the present study, we assessed mental health stigma broadly, rather than specific to each disorder. Therefore, it is possible that PCPs did not necessarily endorse greater stigma towards OCD-Order relative to OCD-Aggression, but rather stigma was a more important contributor to the mislabeling for OCD-Order, specifically. This is in line with research on trivialization in OCD, which shows that more commonly known symptoms of OCD, such as contamination and order, are largely trivialized [70], rather than stigmatized [71]. Therefore, it is possible that PCPs with higher levels of stigma were more likely to trivialize OCD-Order symptoms, and thereby, not label it with the proper diagnosis.

PCPs who reported having a family member/friend with a mental health condition were significantly more likely to recognize SAD. This is similar to research on undergraduate students that found that having a friend with a mental health condition significantly increased the likelihood of recognizing SAD and GAD, but not OCD, PD, or MDD [18]. As SAD is often underrecognized or misdiagnosed in primary care settings [72, 73], it is possible that family members or friends may be more attuned to the specific symptoms of SAD. This may be different than conditions such as GAD and MDD, where recognition rates are generally higher than SAD (e.g [72, 74, 75]), or OCD, where disclosure to family members or friends may be less common [76] and may be associated with greater rejection [44] relative to SAD.

The present study identifies limitations and strengths in the knowledge of mental health conditions among PCPs in Hungary. The present study highlights specific populations (e.g., older physicians) that may be more likely to misdiagnose mental health conditions. However, as our data is cross-sectional and not nationally representative, generalizations about the mental health literacy of PCPs across Hungary and the demographic features that predict accurate identification of mental health conditions should be interpreted with care. Other factors, such as social desirability bias and internet-based data collection (i.e., allowing physicians to use search engines to research the answers) could have confounded our findings. Moreover, as participation was voluntary and participants were not compensated, it is probable that the present study had a selection bias, whereby PCPs with greater interest and education in mental health were more likely to participate, inflating our recognition rate. Additionally, in the present study, the most commonly endorsed primary cause for each condition was mental illness. These favorable findings may be because our survey did not include any physical health conditions; thereby, it is possible that participants recognized that every vignette presented a mental health condition. Future research designs should consider integrating both physical and mental health conditions to determine participants’ ability to distinguish between presentations, as this is more representative of how patients present in real life. Lastly, several additional variables, such as PCP specialty and training experience, may be important predictors of disorder recognition and treatment recommendations, however, these were not assessed in present study. Future research would benefit from a more comprehensive assessment of PCP-related predictors to better inform what contributes to gaps in mental health literacy.

## Conclusions

These limitations notwithstanding, our findings add to the literature by outlining potential intervention targets (e.g., increasing education on appropriate anxiolytic use) to improve mental health literacy in primary care. Future research should investigate the efficacy of psychoeducation interventions, particularly for OCD and anxiety disorders, in improving the mental health literacy of PCPs in Hungary.

## Electronic supplementary material

Below is the link to the electronic supplementary material.

Supplementary Material 1

## Data Availability

Data will be made available upon request from the corresponding author.

## Abbreviations

PCP: Primary Care Provider
OCD: Obsessive-Compulsive Disorder
OCD-Order: Obsessive-Compulsive Disorder with symptoms related order/symmetry
OCD-Aggression: Obsessive-Compulsive Disorder with symptoms related to harm/aggression
GAD: Generalized Anxiety Disorder
SAD: Social Anxiety Disorder
PD: Panic Disorder
MDD: Major Depressive Disorder
NEAK: National Healthcare Fund in Hungary
OMS-HC: Opening Minds Stigma Scale for Health Care Providers
