# Supplementary material for: Mental health literacy among primary care providers in Hungary: a vignette-based survey
Source: Ann Gen Psychiatry. 2025 Jan 28;24:6. doi: 10.1186/s12991-024-00539-5 (PMC11776174; doi:10.1186/s12991-024-00539-5)
Supplement: Supplementary file 1 — Supplementary Material 1 [file 12991_2024_539_MOESM1_ESM.docx]

**Appendix A. English Vignettes**

**OCD-Aggression Vignette**: Marcus is 20 years old. He is in the first year of University and frequently experiences thoughts and images with self-aggression content that pop into his head. For example, yesterday while he was having dinner, a thought about hurting and cutting himself with the bread knife popped into his head. Every time he takes the dog for a walk to the vacant lot next to his house and sees a stick on the ground, the thought of hitting his head comes into his mind. He also reports that when he sees sharp objects (e.g., knives or scissors), images of cutting himself come into his head. These thoughts make him feel terrible about himself (he says "it is horrible; I feel terrible whenever these thoughts come into my mind"), and they make it difficult to concentrate on school tasks. He spends a lot of time every day trying to avoid having these thoughts. For example, he constantly looks at his hands to check that there is nothing in them and make sure that he does not hurt himself with objects, a sequence that he repeats mentally to check that he has done nothing. He tries to feel better by avoiding using knifes or walking the dog; and he tries to convince himself that he is not going to hurt himself by saying: "I do not want to hurt myself", and tries to suppress or control these thoughts by saying "I have to stop thinking about these strange things ...".

**OCD-Order Vignette:** John is a 23-year-old student. For the past 7 months, he has felt the need to have things arranged in a certain way because if they are not placed in a certain way, he feels really anxious and afraid that something bad will happen to him or his family. To feel better, he performs a number of behaviors that he describes as: "I always have to carry my wallet and mobile phone in the same pocket. I often have to check that everything is in the right place because, if not, I am afraid that something bad will happen. To be able to study, it is essential for everything to be tidy, with fully aligned books, the case to the left of the notes, and the window blind fully raised. Also, in the kitchen, cutlery, glasses and plates must be perfectly organized by size with no space between them. I know this kind of activity makes no sense, but I cannot avoid doing it, thoughts take control over me, and I spend a lot of time every day trying to avoid bad things that can happen to me". Keeping everything tidy the way he likes it makes him waste more than 2 h a day. Because of these things that might happen to him, he lives in a state of constant tension, which makes it difficult to sleep and makes him irritable all the time.

**SAD Vignette**: John is a 21-year-old living at home with his parents. Since starting college last year, he has become even more shy than usual and has made only one friend. He would really like to make more friends, but he is scared that he will do or say something embarrassing when he’s around others. Although John’s work is OK he rarely says a word in class and becomes incredibly nervous, trembles, blushes and seems like he might vomit if he has to answer a question or speak in front of the class. At home, John is quite talkative with his family, but becomes quiet if anyone he doesn’t know well comes over. He never answers the phone and he refuses to attend social gatherings. He knows his fears are unreasonable but he cannot seem to control them and this really upsets him.

**GAD Vignette**: Susan is 45 years old and she is often worried. She worries a great deal about her job performance, her children’s well being, and her relationships with men. In addition, she worries about a variety of minor matters such as getting to appointments on time, keeping her house clean, and maintaining regular contact with family and friends. It takes Susan longer than necessary to accomplish tasks because she worries about making decisions. Susan has trouble sleeping at night and finds that she is exhausted during the day and irritable with her family.

**PD Vignette**: Derek is 27 years old and he was driving with his wife to a computer store when he felt dizzy. As soon as he noticed this sensation, he experienced a rapid and intense surge of sweating, accelerated heart rate, hot flashes, trembling, and a feeling of detachment from his body. Fearing he was going to crash his car, he pulled off the road. After 10 min the feelings passed and Derek began to feel better, but now he worries extensively that it will happen again and he is reluctant to drive long distances.

**MDD Vignette**: Matt is 30 years old. He has been feeling unusually sad and miserable for the last few weeks. Even though he is tired all the time, he has trouble sleeping nearly every night. Matt does not feel like eating and has lost weight. He cannot keep his mind on his work and puts off making decisions. Even day-to-day tasks seem too much for him. This has come to the attention of his boss, who is concerned about Matt’s lowered productivity.

**Appendix B: Hungarian Translations**

**OCD-Aggression Vignette**: Márk 20 éves. Az egyetem első évfolyamára jár, és gyakran tapasztalja saját magára irányuló erőszakkal kapcsolatos gondolatok és képzetek bevillanását. Tegnap például vacsora közben eszébe villant, hogy megsérti és megvágja magát a kenyérvágó késsel. Valahányszor sétálni viszi a kutyát a háza melletti üres telekre, és meglát egy botot a földön, eszébe villan, hogy megüti vele a fejét. Arról is beszámol, hogy ha éles tárgyakat (pl. kést vagy ollót) lát, akkor olyan képek jutnak az eszébe, hogy megvágja magát. Ezektől a gondolatoktól rettenetesen érzi magát (azt mondja: ”borzalmas; szörnyen érzem magam, amikor ezek a gondolatok eszembe jutnak”), és azok megnehezítik az iskolai feladatokra való koncentrálást. Minden nap sok időt tölt azzal, hogy elhárítsa ezeket a gondolatokat. Például állandóan a kezét nézi, hogy ellenőrizze, hogy nincs benne semmi, és hogy meggyőződjön arról, hogy nem sérti meg magát valamivel. Ezt a sorozatot fejben ismétli, azért, hogy ellenőrizze, nem tett-e semmi effélét. Megpróbálja azzal javítani a közérzetét, hogy kerüli a kés használatát és a kutyasétáltatást. Igyekszik meggyőzni magát arról, hogy nem fog ártani magának, olyan módon, hogy elismétli: ”nem akarok ártani magamnak”. Megpróbálja elnyomni vagy irányítása alá venni ezeket a gondolatokat azzal, hogy „abba kell hagynom ezt a fajta gondolkodást...”.

**OCD-Order Vignette:** János 23 éves diák. Az elmúlt 7 hónapban úgy érezte, hogy bizonyos módon kell elrendezni a dolgokat, mert ha nem egyúgy vannak elhelyezve, akkor nagyon szorong és fél attól, hogy valami rossz történik vele vagy a családjával. Hogy jobban érezze magát, számos cselekvést hajt végre, amelyeket így mutat be: ”Mindig ugyanabban a zsebemben kell hordanom a pénztárcámat és a mobiltelefonomat. Gyakran meg kell néznem, hogy minden a megfelelő helyen van-e, mert ha nem, akkor félek, hogy valami rossz történik. Ahhoz, hogy képes legyen tanulni, az kell, hogy minden rendezett legyen: a könyvek legyenek sorbarendezve, a tok a jegyzetektől balra, teljesen felemelt ablakrolóval. Valamint a konyhában az evőeszközöket, a poharakat és a tányérokat méret szerint tökéletesen el kell rendezni úgy, hogy ne maradjon üres hely közöttük. Tudom, hogy ennek a fajta tevékenységnek nincs értelme, de nem kerülhetem el, a gondolatok átveszik az irányítást felettem, és minden nap sok időt töltök azzal, hogy elkerüljem azokat a rossz dolgokat, amelyek megtörténhetnek velem.” A számára megfelelő rend fenntartására több mint napi 2 órát pazarol el. A félelem miatt, hogy rossz dolog történhetik vele, állandó feszültségben él, ami megnehezíti az alvást és állandóan ingerlékennyé teszi.

**SAD Vignette**: János 21 éves, otthon él a szüleivel. Amióta tavaly elkezdte az egyetemet, a szokásosnál is félénkebb lett, és összesen egy barátot szerzett. Nagyon szeretne még több barátot szerezni, de fél attól, hogy valami kínos dolgot tesz vagy mond, amikor mások közelében van. Bár János jól dolgozik az órán, ritkán szól egy szót is. Hihetetlenül ideges lesz, remeg, elpirul, és hányingere van, ha válaszolnia kell egy kérdésre vagy beszélnie kell az osztály előtt. Otthon János elég beszédes a családjával, de elhallgat, ha valaki megjelenik, akit ő nem ismer jól. Soha nem veszi fel a telefont, és nem hajlandó részt venni a társasági összejöveteleken. Tudja, hogy a félelme ésszerűtlen, de úgy tűnik, nem tudja uralni, és ez nagyon felzaklatja.

**GAD Vignette:** Zsuzsanna 45 éves, gyakran aggódik. Sokat szorong a munkahelyi teljesítménye, a gyermekei jóléte és a férfiakkal való kapcsolata miatt. Ezen túlmenően számos kisebb dolog miatt izgul; például, hogy időben megérkezzen a találkozókra, tisztán tartsa a házát, és rendszeres kapcsolatot tartson fenn családjával és barátaival. Zsuzsannának a szükségesnél tovább tart a feladatok elvégzése, mert aggódik a döntések meghozatala miatt. Zsuzsanna éjszaka nehezen alszik, napközben pedig kimerült, és ingerlékeny a családjával.

**PD Vignette**: Tivadar 27 éves, feleségével egy számítástechinkai üzlet felé autózott, amikor szédülést érzett. Amint észlelte ezt, erős izzadást, felgyorsult szívverést, hőhullámot, reszketést és a testétől való elszakadás érzését tapasztalta. Attól tartva, hogy karambolozik, letért az útról. 10 perc elteltével az érzések elmúltak, és Tivadar kezdte jobban érezni magát, de most nagyon aggódik, hogy ez megismétlődik, és nem szívesen vezet hosszabb távokat.

**MDD Vignette:** Máté 30 éves. Az elmúlt hetekben szokatlanul szomorúnak és nyomorultnak érezte magát. Annak ellenére, hogy állandóan fáradt, szinte minden éjszaka nehezen alszik. Máténak nincs kedve enni, és lefogyott. Nem tudja a munkáját elvégezni, és halogatja a döntéseket. Még a napi feladatok is túl soknak tűnnek számára. Erre a főnöke is felfigyelt, aki aggódik Máté csökkent teljesítménye miatt.

**Appendix C: Question Items**

Is something wrong with the person described in the vignette? That is, what do you think they would be diagnosed with?

- ______________ (single-text entry)

What do you think is the primary cause of this problem?


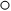
 Mental illness


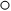
 Stress


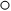
 Biological factors


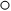
 Environmental Factors


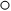
 Personal weakness

- Other _________

Do you think that [name of person in vignette] should seek help for this problem?


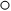
 Yes


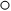
 No

What kind of help would you recommend to [name of person in vignette]? Please rank your choices from 1 to X in order of your preferred recommendation.


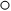
 Therapist or psychologist

- Psychiatrist
- Medication (please specify the type of medication: ______)
- General Practitioner
- Social support (friend, family, teachers)
- Hospital / Emergency Room
- Priest or church
- Concealment
- Other: _______________
